# High Interleukin-6 Plasma Concentration upon Admission Is Predictive of Massive Transfusion in Severely Injured Patients

**DOI:** 10.3390/jcm10112268

**Published:** 2021-05-24

**Authors:** Nadja Weichselbaum, Daniel Oberladstätter, Christoph J. Schlimp, Johannes Zipperle, Wolfgang Voelckel, Oliver Grottke, Georg Zimmermann, Marcin Osuchowski, Herbert Schöchl

**Affiliations:** 1 Department of Anaesthesiology and Intensive Care Medicine, AUVA Trauma Centre Salzburg, Academic Teaching Hospital of the Paracelsus Medical University, 5020 Salzburg, Austria; nadja.weichselbaum@stud.pmu.ac.at (N.W.); daniel.oberladstaetter@auva.at (D.O.); Wolfgang.voelckel@auva.at (W.V.); 2 Paracelsus Medical University, 5020 Salzburg, Austria; 3 Ludwig Boltzmann Institute for Experimental and Clinical Traumatology, AUVA Trauma Research Centre, 1020 Vienna, Austria; christoph.schlimp@trauma.lbg.ac.at (C.J.S.); johannes.zipperle@trauma.lbg.ac.at (J.Z.); marcin.osuchowski@trauma.lbg.ac.at (M.O.); 4 Department of Anaesthesiology and Intensive Care Medicine, AUVA Trauma Centre Linz, 4010 Linz, Austria; 5 Department of Anaesthesiology, RWTH Aachen University Hospital, 52074 Aachen, Germany; ogrottke@ukaachen.de; 6 Team Biostatistics and Big Medical Data, IDA Lab Salzburg, Paracelsus Medical University, 5020 Salzburg, Austria; georg.zimmermann@pmu.ac.at; 7 Department of Research and Innovation, Paracelsus Medical University, 5020 Salzburg, Austria

**Keywords:** interleukin-6, coagulopathy, trauma, massive transfusion

## Abstract

Severe bleeding remains a prominent cause of early in-hospital mortality in major trauma patients. Thus, prompt prediction of patients at risk of massive transfusion (MT) is crucial. We investigated the ability of the inflammatory marker interleukin (IL)-6 to forecast MT in severely injured trauma patients. IL-6 plasma levels were measured upon admission. Receiver operating characteristic curves (ROCs) were calculated, and sensitivity and specificity were determined. In this retrospective study, a total of 468 predominantly male (77.8%) patients, with a median injury severity score (ISS) of 25 (17–34), were included. The Youden index for the prediction of MT within 6 and 24 h was 351 pg/mL. Patients were dichotomized into two groups: (i) low-IL-6 < 350 pg/mL and (ii) high-IL-6 ≥ 350 pg/mL. IL-6 ≥ 350 pg/mL was associated with a lower prothrombin time index, a higher activated partial thromboplastin time, and a lower fibrinogen concentration compared with IL-6 < 350 pg/mL (*p* <0.0001 for all). Thromboelastometric parameters were significantly different between groups (*p* <0.03 in all). More patients in the high-IL-6 group received MT (*p* <0.0001). The ROCs revealed an area under the curve of 0.76 vs. 0.82 for the high-IL-6 group for receiving MT in the first 6 and 24 h. IL-6 ≥ 350 pg/mL predicted MT within 6 and 24 h with a sensitivity of 45% and 58%, respectively, and a specificity of 89%. IL-6 ≥ 350 pg/mL appears to be a reasonable early predictor for coagulopathy and MT within the first 6 and 24 h intervals. Large-scale prospective studies are warranted to confirm these findings.

## 1. Introduction

Exsanguination remains a leading but potentially preventable cause of early in-hospital mortality in severely injured patients [1]. Approximately one-quarter to one-third of all major trauma patients suffer from uncompressible, microvascular bleeding, termed trauma-induced coagulopathy (TIC) [2,3,4]. TIC has been identified as an independent predictor of poor outcome. Coagulopathic trauma patients have a higher bleeding tendency, higher transfusion requirements, and an almost four-fold increase in mortality compared with similarly injured patients without hemostatic alterations [2].

In addition to the quick detection of the underlying coagulopathy, the rapid estimation of the risk of massive transfusion (MT) is warranted. A variety of MT prediction scores have been developed so far [5,6,7,8]. In general, the positive predictive values of these scores are limited [9]. Given that severe tissue trauma is a potent trigger for the upregulation of inflammatory cytokines [10], several of those mediators were investigated for their predictive capacity regarding various post-traumatic outcomes. Interleukin (IL)-6 has received increasing interest as the most promising candidate [11,12,13,14,15]. Several studies revealed that high circulating IL-6 in trauma patients was associated with multi-organ dysfunction syndrome (MODS), acute respiratory distress syndrome (ARDS), and mortality [12,16,17].

Although intensive crosstalk between inflammation and coagulation has been well-described for sepsis, less is known about the impact of IL-6 on coagulopathy and transfusion requirements in major trauma [18,19]. Data from the Inflammation and the Host Response to Injury Large Scale Collaborative Program revealed that elevation of circulating IL-6 was not only associated with MODS but also with MT [16]. However, IL-6 measurements in that study were performed at a random time point within 12 h after hospital admission. In our hospital, IL-6 is routinely measured upon emergency room (ER) admission in severely injured patients requiring full trauma team activation.

The primary objective of this retrospective study was to evaluate the association between the circulating IL-6 and blood transfusion requirements. We specifically hypothesized that in trauma patients, IL-6 blood concentration measured upon ER admission is associated with coagulopathy and predicts the need for an MT.

## 2. Materials and Methods

### 2.1. Study Design

**Figure 1:**
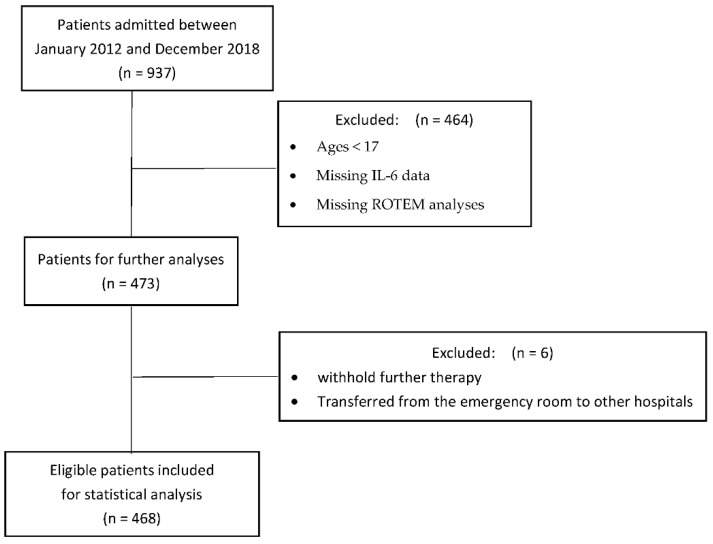
Flow chart of included and excluded patients.

Following the local ethics committee approval (AUVA EK 08/2020), we performed a retrospective analysis of trauma patients admitted between January 2012 and December 2018 to the AUVA Trauma Center Salzburg, Austria, a certified level 1 Trauma Hospital. All patients >17 years in whom IL-6 and rotational thromboelastometry (ROTEM) measurements were performed upon ER admission were eligible for analysis. Exclusion criteria were patients <17 years, patients missing IL-6 and ROTEM data, patients transferred from other hospitals and transferred from our ER in other facilities, and patients in whom further therapy was withheld due to futility. A flow chart outlines included and excluded patients (Figure 1).

Since 2012, prospective, standardized, and anonymous documentation of all major trauma patients admitted to the AUVA Trauma Centre has been established, and these data were forwarded to the central database of the TraumaRegister DGU^®^ (TR-DGU) of the German Trauma Society (Deutsche Gesellschaft für Unfallchirurgie, DGU). This documentation was used for analysis, and missing variables were extracted from the anesthesia and intensive care unit (ICU) database (COPRA 6) and the electronic patient information system (ASTRA).

### 2.2. Laboratory Measurements, Standard Coagulation Tests, and ROTEM Analyses

Upon ER admission, the following laboratory measurements are performed in major trauma patients on a routine basis: full blood cell count with differential and standard coagulation parameters, including fibrinogen concentration (Clauss method, normal range: 200 to 450 mg/dL); prothrombin time index (PTI, normal range: 70–130%), activated partial thromboplastin time (aPTT, normal range: 23.7 to 34.9 s); and ROTEM analysis. From January 2015 to June 2016, EXTEM, INTEM, and FIBTEM analyses were run on a ROTEM^®^ delta device (TEM International, Munich, Germany); from July 2016 onwards, a fully automated cartridge-based ROTEM^®^ sigma device has been used. Moreover, arterial blood gas analyses were used to quantify base excess (BE, normal range: −3.0 to +3.0 mmol/L) and lactate concentration (normal range: 1 to 1.8 mmol/L).

IL-6 (normal range: 0–7 pg/mL) measurement is also part of our standard laboratory panel upon ER admission of severely injured patients.

### 2.3. Coagulation Therapy and Allogeneic Blood Transfusion

Coagulation therapy was primarily based on coagulation factor concentrates and guided by ROTEM test results [20]. Hemostatic therapy and transfusion requirements for the individual patients were obtained from the electronic trauma databases of the hospital (COPRA-6^®^, COPRA System GmbH, Berlin, Germany) and a special database where all allogeneic blood transfusions have to be documented (DataLab^®^, Bartelt GmbH, Graz, Austria). Massive transfusion was defined as a red blood cell (RBC) transfusion ≥6 U within 6 h or ≥10 RBC/24 h [21].

### 2.4. Statistical Analysis

Distribution of the data was assessed using the Shapiro–Wilk test. Continuous variables are expressed as median and interquartile ranges (25th percentile, 75th percentile). Categorical variables are reported as numbers and percentages (%), and compared between groups using Fisher’s exact or chi-squared tests. For analyzing the between-group differences in the metric variables, the Mann–Whitney U test was used. The sensitivity and specificity of the analyzed data were calculated by receiver operating characteristic (ROC) curves, and predictive capacity is expressed by the area under the curve (AUC) value. The optimal IL-6 level for prediction of MT at 6 and 24 h was determined by Youden’s index (sensitivity + specificity − 1)

Multiple logistic regression models were fitted to the data to adjust for potential associations with other variables. In the first step, a list of several potential variables were specified. Subsequently, the coefficients/odds ratios were estimated, using an IL-6 value of ≥350 pg/mL as a cutoff for the dichotomization of the outcome, and a forward variable selection procedure was applied.

Statistical calculations were performed using GraphPad Prism (Version 9.0.0, Graph-Pad Software, La Jolla, CA, USA) and R statistics (R Core Team 2019). *p*-values below 0.05 were considered significant [22].

## 3. Results

A total of 468 predominantly (77.8%) male patients were included in the current study. The median age of the patients was 49 (33.3–64.0) years, the Injury Severity Score (ISS) was 25 (17–34), and the new ISS (NISS) was 29 (22–43). The median intensive care unit (ICU) length of stay was 9 (4–16) days. A total of 29 (6.2%) patients received MT and 50 (10.6%) died.

The Youden’s index calculation for the prediction of massive transfusion within 6 and 24 h was 351 pg/mL. According to previous reports [16], we used an IL-6 cutoff value of 350 pg/mL and dichotomized the patients into two groups for further analyses: (i) low-IL-6 < 350 pg/mL and (ii) high-IL-6 ≥ 350 pg/mL.

### 3.1. Demographic and Clinical Data

The abbreviated injury score (AIS) for most body regions was significantly higher in patients in the high-IL-6 group compared with the low-IL-6 group. Upon ER admission, patients in the high-IL-6 group were in a more severe shock state compared with the low-IL6 group, as indicated by significantly lower systolic blood pressure, a higher heart rate, and a lower hemoglobin concentration upon admission. This is also reflected by a lower base excess and a higher lactate concentration (*p* < 0.001 for all). Table 1 outlines demographic and clinical parameters recorded upon hospital admission.

### 3.2. Laboratory Data

Importantly, high-IL-6 patients were frequently more coagulopathic upon ER admission compared with low-IL-6 patients (Table 2). Both standard coagulation tests and ROTEM parameters were significantly different for all the investigated measurements between groups (*p* < 0.05 for all).

### 3.3. Coagulation Therapy and Transfusion Requirements

**Figure 2:**
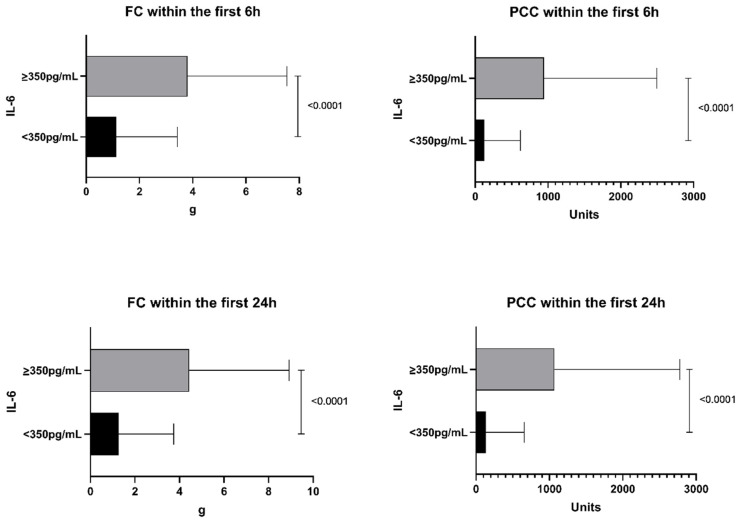
Coagulation therapy of patients in the high-IL-6 group (≥350 pg/mL) and the low-IL-6 group (<350 pg/mL)). IL-6, interleukin 6; FC, fibrinogen concentrate; PCC, prothrombin complex concentrate.

**Figure 3:**
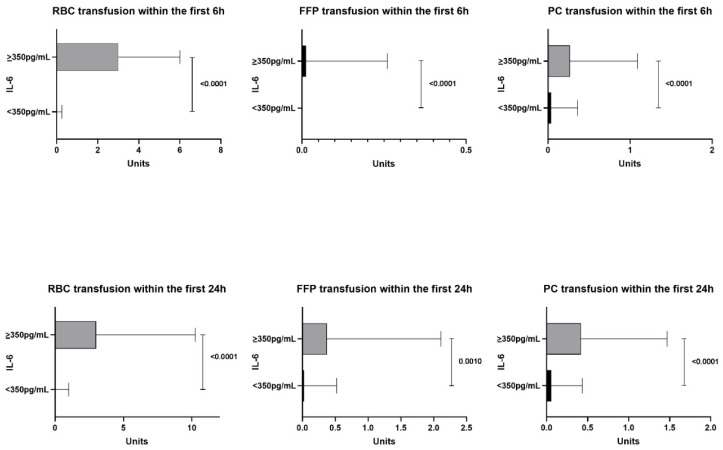
RBC, FFP and platelet concentrate transfusion of allogeneic blood products in the high-IL-6 group (≥350 pg/mL) and the low-IL-6 group (<350 pg/mL). IL-6, interleukin 6; RBC, red blood cells; FFP, fresh frozen plasma; PC, platelet concentrates.

Coagulopathy was also evident, given that patients in the high-IL-6 group received significantly higher amounts of fibrinogen concentrate and prothrombin complex concentrate within the first 6 and 24 h compared with the low-IL-6 group (*p* < 0.05 for all; Figure 2). The transfusion of allogeneic blood products is outlined in Figure 3. Significant differences were observed for all allogeneic blood transfusions for the first 6 and 24 h after ER admission.

### 3.4. ROC Analyses

**Figure 4:**
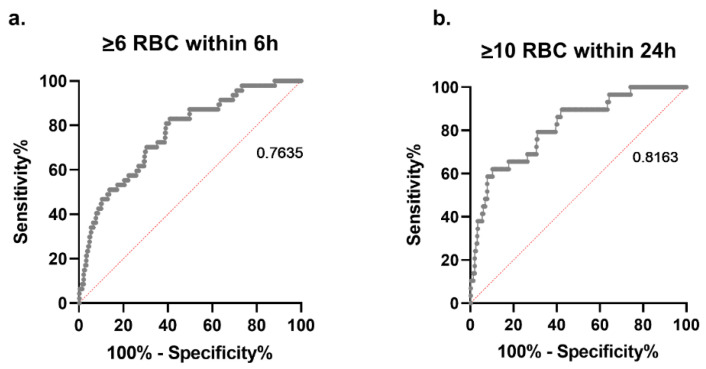
Receiver operating characteristic curve with corresponding area under the curve for serum IL-6 levels ≥350 pg/mL, and massive transfusion within (**a**) the first 6 h (≥6 RBCs) and (**b**) 24 h (≥10 RBCs) upon emergency room admission.

The ROC for transfusion of ≥6 RBCs within the first 6 h after hospital admission revealed an AUC of 0.77 and for transfusion of ≥10 RBCs within 24 h, an AUC of 0.82 was revealed (Figure 4).

Sensitivity, specificity, and the positive and negative predictive value of IL-6 ≥ 350 pg/mL regarding MT within 6 and 24 h are outlined in Table 3.

### 3.5. Multiple Logistic Regression Analyses

To identify confounding factors for high IL-6 levels, a multiple logistic regression model was performed. A positive association with IL-6 ≥350 pg/mL was identified for the NISS, the time from injury to hospital admission, and RBC transfusion within the first 24 h. A negative association was observed for prothrombin time index (PTI) (Table 4).

## 4. Discussion

To the best of our knowledge, this is the first study that investigated the association of IL-6 plasma concentration upon ER admission with coagulopathy and allogeneic blood transfusion requirements in a cohort of severely injured trauma patients. The current study revealed that IL-6 is a valid early prognostic marker for MT. IL-6 ≥ 350 pg/mL upon hospital admission predicted MT within 6 and 24 h with a sensitivity and specificity comparable to previously published MT prediction scores [16].

Patients with severe trauma upregulate both pro- and anti-inflammatory cytokines [23] and IL-6 has received the most interest regarding its general predictive potential. Damaged tissues and cells such as monocytes, T and B lymphocytes, and endothelial cells rapidly synthesize/release IL-6 following the trauma event and stimulation by other cytokines [15]. Some studies have demonstrated the usefulness of the circulating IL-6 measurement after traumatic injuries to predict MODS, sepsis, and mortality [12,16,24,25]. However, less is known regarding the post-traumatic capacity of IL-6 fluctuations to predict coagulopathy, hemostatic therapy, and transfusion requirements.

The quantity of IL-6 released into the blood stream is primarily related to the extent of the tissue trauma and, to a lesser degree, to the severity and duration of shock [11,26]. Patients in the high-IL-6 group (vs. low IL-6) were significantly more injured as reflected by higher median ISS, NISS, and AIS. This finding is consistent with that of Gebhard et al., who reported a correlation between IL-6 level and the extent of tissue trauma indicated by ISS [25]. Compared with patients with ISS < 18, the subjects with an ISS > 18 not only had an elevated IL-6 upon ER admission, but also revealed a substantial increase in circulating IL-6 over the following 6 h [26].

In contrast with trauma, the impact of hemorrhagic shock alone on IL-6 release remains controversial. Roumen et al. observed that the occurrence of shock has only a minor impact on IL-6 generation [27]. Conversely, Halbgebauer et al. reported a significantly higher IL-6 concentration in polytrauma patients with hemorrhagic shock compared with those suffering from multiple traumas without an accompanying shock [28]. In clinical practice, base excess and lactate are often used as surrogates for the severity of shock [29]. In the current study, the median BE in the high-IL-6 group was significantly lower compared with the low-IL-6 group.

Patients with an established TIC upon ER admission are prone to higher complication rates and increased mortality compared with patients without coagulopathy [2,3]. It is well-known that inflammation and coagulation are tightly interwoven [18,19]. However, it has not yet been established whether the post-traumatic changes in circulating IL-6 are associated with coagulopathy. In animal models, IL-6 infusions elevated PT, aPTT, and bleeding time [30]. In cancer patients, infusions of recombinant human IL-6 significantly increased activation markers of coagulation such as thrombin-antithrombin III complexes and prothrombin fragment F1 + 2, without substantially impacting fibrinolysis [31]. The role of IL-6 as a mediator of hemostatic change during severe inflammation is controversial. In the current study, PTI and fibrinogen levels were markedly lower, and aPTT was significantly prolonged in the high-IL-6 compared with the low-IL-6 group. The multivariate analyses revealed a negative correlation between PTI and IL-6. Viscoelastic test results, in particular the clot amplitude after a 10 min running time, were also significantly different between the low- and high-IL-6 groups. A diminished early clot amplitude is increasingly being used to define coagulopathy and identify patients at risk for MT [20,32]. Our group calculated an FIBTEM MCF value < 7 mm upon ER admission to provide early prediction of MT (≥10 RBCs/24 h), and reported an ROC AUC of 0.84 [33]. The ROC AUC for the prediction of MT in the current study revealed only a slightly lower value (AUC 0.82) for IL-6 ≥ 350 pg/mL.

An early prediction of MT upon ER admission is highly warranted, but remains challenging. Brockamp et al. validated six different MT prediction scores and reported only limited sensitivity and specificity [9]. The highest prognostication accuracy for MT was observed for the trauma associated severe hemorrhage (TASH) score, with an AUC of 0.89 [5]. The TASH score is laborious to calculate; therefore, its acceptance in clinical practice is limited. The cumulative sensitivity and specificity of IL-6 ≥350 pg/mL for prediction of MT within the first 24 h after ER admission is comparable to the TASH score. Moreover, we calculated a higher PPV for MT than the TASH score (PPV 24.2% vs. 18.9%). Another frequently used prognostication score for MT is the assessment of blood consumption (ABC) score, which was applied in the PROPPR study, a large prospective randomized trial investigating different ratios of RBCs to fresh frozen plasma [34]. In the Brokamp validation study, the ROC AUC for the ABC score was 0.76, which is similar to our 6 h MT prediction in the high-IL-6 group but substantially lower than our 24 h MT prediction (AUC 0.82) [9].

### Limitations

There are several limitations in the current study, which must be considered when interpreting its findings. First, the analyses were performed retrospectively, and our study suffers from the inherent shortcomings of such a data evaluation.

Second, IL-6 concentration in the blood is not only related to the severity of tissue trauma but also to a time-dependent variable. Thus, the time elapsed between the injury and hospital admission is a crucial factor as IL-6 typically increases over a short time period [15]; it takes approximately 1 h to detect circulating IL-6 after trauma [35]. We acknowledged this association in our multivariate analysis by displaying a positive correlation between IL-6 levels and the time to the emergency room admission. Of note, in patients with a severe hemorrhagic traumatic shock and a rapid transport time, the predictive value of IL-6 may be limited due to its still relatively low circulating concentration upon admission.

Third, hemostatic therapy in our hospital is primarily based on coagulation factor concentrates. Small prospective and retrospective studies revealed that this strategy might result in a lower incidence in MT compared with a plasma-based coagulation therapy [36,37,38]. Thus, our findings have to be confirmed in trauma patients receiving high-volume plasma therapy.

Forth, in contrast with point-of-care devices, such as viscoelastic and blood gas analyzers, IL-6 measurements are time consuming. Thus, results for decision making are available only with considerable time delay.

## 5. Conclusions

High IL-6 level upon emergency room admission is associated with a substantial impairment of hemostasis. Trauma patients with plasma IL-6 exceeding 350 pg/mL are prone to MT. Sensitivity and specificity, as well as positive and negative predictive values, are comparable to the current MT prediction scores. Our findings support the use of IL-6 measurements upon ER admission as a fast laboratory parameter to identify coagulopathic trauma patients with the potential need for MT. However, large-scale studies are warranted to confirm our findings.

## Figures and Tables

**Table 1:** Demographics, clinical data, and injury scores upon emergency room admission.

	All Patients	IL6 < 350 pg/mL	IL6 ≥ 350 pg/mL	*p*-Value
Number	468	406	62	
Age (years)	49 (33–64)	47 (31–57)	50 (34–65)	
Male (n, %)	364 (77.8)	313 (77.1)	51 (82.3)	
Heart rate (bpm)	90 (75–105)	88 (73–102)	102 (83–129)	<0.0001
Systolic BP (mmHg)	125 (104–145)	127 (108–148)	107 (68–125)	<0.0001
**Prehospital fluid therapy**				
Crystalloids (mL)	500 (500–1000)	500 (500–1000)	1000 (500–1313)	0.0002
Colloids (mL)	0 (0–0)	0 (0–0)	0 (0–500)	0.0004
Time from injury to ER (min)	68 (54–95)	64 (52–90)	99 (73–144)	<0.0001
**RBC transfusion**				
≥6 RBCs/6 h (n, %)	47 (10.0)	20 (4.9)	27 (43.5)	<0.0001
≥10 RBCs/24 h (n, %)	29 (6.2)	12 (1.7)	17 (27.4)	<0.0001
Length of ICU stay (days)	9 (4–16)	8 (4–16)	14 (5.5–23)	0.0013
Mortality (n, %)	50 (10.7)	38 (9.4)	12 (19.4)	0.0264
**Injury-Scores**				
ISS	25 (17–34)	25 (17–33)	38 (25–49)	<0.0001
NISS	29 (22–43)	29 (20–41)	43 (30–57)	<0.0001
AIS Head	2 (0–4)	2 (0–4)	2 (0–4)	ns
AIS Face	0 (0–0)	0 (0–0)	0 (0–0)	ns
AIS Thorax	3 (0–3)	2 (0–3)	3 (3–5)	<0.0001
AIS Abdomen	0 (0–2)	0 (0–2)	2 (0–3)	<0.0001
AIS Extremities and Pelvis	2 (0–3)	2 (0–3)	2 (2–4)	0.0024
AIS Soft Tissue	0 (0–0)	0 (0–0)	0 (0–0)	ns
TASH	5 (2–8)	4 (2–7)	11 (6–15)	<0.0001

Abbreviations: IL-6, interleukin 6; RBC, red blood cells; BP, blood pressure; ISS, injury severity score; NISS, new injury severity score; AIS, abbreviated injury score; TASH, trauma associated severe hemorrhage score; ns, not significant.

**Table 2:** Circulating IL-6, and selected blood and coagulation measures upon ER admission.

Laboratory-Data	All Patients	IL6 < 350 pg/mL	IL6 ≥ 350 pg/mL	*p* Value
IL-6 (pg/mL)	91.15 (35–215.2)	67 (28.25–137.2)	511.3 (411.5–944.5)	<0.0001
Hemoglobin (g/dL)	13.1 (11.8–14.3)	13.3 (12–14.5)	11.7 (9.9–13.45)	<0.0001
Platelet count (10^3^/µL)	222 (187–264)	223 (188–264)	220 (180–261)	ns
pH	7.35 (7.29–7.39)	7.36 (7.13–7.39)	7.25 (7.16–7.33)	<0.0001
Base-excess (mmol/L)	2.5 (−4.7–−1.0)	−2.3 (–4.1–−0.82)	−6.5 (−11.4–−3.8)	<0.0001
Lactate (mmol/L)	2.2 (1.4–3.4)	2.1 (1.4–3)	4.2 (2.3–8.8)	<0.0001
**Standard coagulation tests**				
PTI (%)	89 (73–100)	91 (79–102)	64 (50.25–77)	<0.0001
aPTT (s)	27 (25–30)	27 (25–29)	33 (28–39)	<0.0001
Fibrinogen (mg/dL)	245 (197–298)	254 (208–302)	185 (140–243)	<0.0001
**ROTEM parameters:**				
EXTEM				
Clotting time (s)	63 (56–77)	63 (56–75)	70 (59–88)	0.0198
Clot formation time (s)	113 (90–139)	112 (89–134)	131 (98–157)	0.0045
Alpha (°)	70 (65–74)	70 (65–74)	67 (62–73)	0.0272
Maximum clot firmness (mm)	59 (55–63)	59 (56–63)	56.5 (53.3–63.8)	0.0282
Amplitude 10 min (mm)	51 (46–55)	52 (46–55)	48 (41–54)	0.0032
Lysis index 45 (%)	99 (97–100)	98 (97–99)	99 (98–100)	0.0004
**INTEM**				
Clotting time (s)	157 (143–172)	157 (143–170)	165 (145–198)	0.0049
Clot formation time (s)	80 (66–95)	79 (65–92)	90 (72–14)	0.0012
Alpha (°)	74 (71–77)	75 (72–77)	72 (68–75)	0.0007
Maximum clot firmness (mm)	61 (57–64)	61 (57–64)	59 (54–64)	0.0235
Amplitude 10 min (mm)	54 (50–58)	55 (50–59)	52 (46–55)	0.0005
Lysis index 45 (%)	97 (96–99)	97 (95–98,25)	99 (97–100)	<0.0001
**FIBTEM**				
Clotting time (s)	64 (57–76)	63 (57–74)	70 (60–87)	0.0257
Maximum clot firmness (mm)	12 (8–15)	12 (9–15)	9 (7–14)	0.0022
Amplitude 10 min (mm)	11 (8–14)	12 (8–14)	9 (6–13)	0.0002

Abbreviations: IL-6, interleukin 6; PTI, prothrombin time index; aPTT, activated partial thromboplastin time; ROTEM, rotational thromboelastometry; EXTEM, extrinsically activated assay; INTEM, intrinsically activated assay; FIBTEM, fibrin-polymerization assay.

**Table 3:** Massive transfusion prediction of IL-6 > 350 pg/mL.

	Sensitivity (%) for IL-6 ≥ 350 pg/mL	Specificity (%) for IL-6 ≥ 350 pg/mL	PPV (%) for IL-6 ≥ 350 pg/mL	NPV (%) for IL-6 ≥ 350 pg/mL
≥6 RBC/6 h	45.2 (27.3–64.0)	89.0 (85.7–91.8)	22.6	95.8
≥10 RBC/24 h	57.7 (36.9–76.6)	89.4 (86.1–92.1)	24.2	97.3

IL-6, interleukin 6; PPV, positive predictive value; NPV, negative predictive value; RBC, red blood cells.

**Table 4:** Multivariate analysis for IL-6 values < 350 pg/dL vs. ≥ 350 pg/dL.

	Odds Ratio	95% CI	z-Value	*p*-Value
Time from injury to ER (min)	1.01	(1.00–1.01)	3.789	0.0002
NISS	1.03	(1.01–1.05)	2.449	0.0143
PTI (%)	0.97	(0.96–0.99)	−3.375	0.0007
RBCs first 24 h	1.1	(1.0–1.2)	2.940	0.0033

ER, emergency room; NISS, new injury severity score; PTI, prothrombin time index; RBC, red blood cells.

## Data Availability

The data presented in this study are available on request from the corresponding author.

## References

[1] Kauvar D.S., Lefering R., Wade C.E. (2006). Impact of Hemorrhage on Trauma Outcome: An Overview of Epidemiology, Clinical Presentations, and Therapeutic Considerations. J. Trauma Acute Care Surg..

[2] Brohi K., Singh J., Heron M., Coats T. (2003). Acute Traumatic Coagulopathy. J. Trauma Acute Care Surg..

[3] MacLeod J.B.A., Lynn M., McKenney M.G., Cohn S.M., Murtha M. (2003). Early Coagulopathy Predicts Mortality in Trauma. J. Trauma Acute Care Surg..

[4] Khan S., Brohi K., Chana M., Raza I., Stanworth S., Gaarder C., Davenport R. (2014). Hemostatic resuscitation is neither hemostatic nor resuscitative in trauma hemorrhage. J. Trauma Acute Care Surg..

[5] Yucel N., Lefering R., Maegele M., Vorweg M., Tjardes T., Ruchholtz S., Neugebauer E.A.M., Wappler F., Bouillon B., Rixen D. (2006). Trauma Associated Severe Hemorrhage (TASH)-Score: Probability of Mass Transfusion as Surrogate for Life Threatening Hemorrhage after Multiple Trauma. J. Trauma Acute Care Surg..

[6] Vandromme M.J., Griffin R.L., Kerby J.D., McGwin G., Rue L.W., Weinberg J.A. (2011). Identifying risk for massive transfusion in the relatively normotensive patient: Utility of the prehospital shock index. J. Trauma Acute Care Surg..

[7] Callcut R.A., Cripps M.W., Nelson M.F., Conroy A.S., Robinson B.B., Cohen M.J. (2016). The Massive Transfusion Score as a decision aid for resuscitation: Learning when to turn the massive transfusion protocol on and off. J. Trauma Acute Care Surg..

[8] Cotton B.A., Dossett L., Haut E.R., Shafi S., Nunez T.C., Au B.K., Zaydfudim V., Johnston M., Arbogast P., Young P.P. (2010). Multicenter Validation of a Simplified Score to Predict Massive Transfusion in Trauma. J. Trauma Acute Care Surg..

[9] Brockamp T., Nienaber U., Mutschler M., Wafaisade A., Peiniger S., Lefering R., Bouillon B., Maegele M., DGU T. (2012). Predicting on-going hemorrhage and transfusion requirement after severe trauma: A validation of six scoring systems and algorithms on the TraumaRegister DGU^®^. Crit. Care.

[10] Giannoudis P., Hildebrand F., Pape H.C. (2004). Inflammatory serum markers in patients with multiple trauma. Can they predict outcome?. J. Bone Jt. Surg. Br. Vol..

[11] Biffl W.L., Moore E.E., Moore F.A., Peterson V.M. (1996). Interleukin-6 in the injured patient. Marker of injury or mediator of inflammation?. Ann. Surg..

[12] Frink M., van Griensven M., Kobbe P., Brin T., Zeckey C., Vaske B., Krettek C., Hildebrand F. (2009). IL-6 predicts organ dysfunction and mortality in patients with multiple injuries. Scand. J. Trauma Resusc. Emerg. Med..

[13] Hirsiger S., Simmen H.-P., Werner C.M.L., Wanner G.A., Rittirsch D. (2012). Danger Signals Activating the Immune Response after Trauma. Mediat. Inflamm..

[14] Okeny P.K., Ongom P., Kituuka O. (2015). Serum interleukin-6 level as an early marker of injury severity in trauma patients in an urban low-income setting: A cross-sectional study. BMC Emerg. Med..

[15] Jawa R.S., Anillo S., Huntoon K., Baumann H., Kulaylat M.N. (2010). Interleukin-6 in Surgery, Trauma, and Critical Care Part II: Clinical Implications. J. Intensiv. Care Med..

[16] Cuschieri J., Bulger E., Schaeffer V., Sakr S., Nathens A.B., Hennessy L., Minei J., Moore E.E., O’Keefe G., Sperry J. (2010). Early elevation in random plasma IL-6 after severe injury is associated with development of organ failure. Shock.

[17] Qiao Z., Wang W., Yin L., Luo P., Greven J., Horst K., Hildebrand F. (2018). Using IL-6 concentrations in the first 24 h following trauma to predict immunological complications and mortality in trauma patients: A meta-analysis. Eur. J. Trauma Emerg. Surg..

[18] Esmon C.T. (2003). Coagulation and inflammation. J. Endotoxin Res..

[19] Huber-Lang M., Sarma J.V., Zetoune F.S., Rittirsch D., Neff T.A., McGuire S.R., Lambris J.D., Warner R.L., Flierl M.A., Hoesel L.M. (2006). Generation of C5a in the absence of C3: A new complement activation pathway. Nat. Med..

[20] Schöchl H., Voelckel W., Schlimp C.J. (2014). Management of traumatic haemorrhage—The European perspective. Anaesthesia.

[21] Zatta A.J., McQuilten Z.K., Mitra B., Roxby D.J., Sinha R., Whitehead S., Dunkley S., Kelleher S., Hurn C., Cameron P.A. (2014). Elucidating the clinical characteristics of patients captured using different definitions of massive transfusion. Vox Sang..

[22] R Core Team (2018). R: A Language and Environment for Statistical Computing.

[23] Ciriello V., Gudipati S., Stavrou P.Z., Kanakaris N.K., Bellamy M.C., Giannoudis P.V. (2013). Biomarkers predicting sepsis in polytrauma patients: Current evidence. Injury.

[24] Volpin G., Cohen M., Assaf M., Meir T., Katz R., Pollack S. (2014). Cytokine Levels (IL-4, IL-6, IL-8 and TGFβ) as Potential Biomarkers of Systemic Inflammatory Response in Trauma Patients. Int. Orthop..

[25] Billeter A., Turina M., Seifert B., Mica L., Stocker R., Keel M. (2009). Early Serum Procalcitonin, Interleukin-6, and 24-Hour Lactate Clearance: Useful Indicators of Septic Infections in Severely Traumatized Patients. World J. Surg..

[26] Gebhard F., Pfetsch H., Steinbach G., Strecker W., Kinzl L., Brückner U.B. (2000). Is Interleukin 6 an Early Marker of Injury Severity Following Major Trauma in Humans?. Arch. Surg..

[27] Roumen R.M.H., Hendriks T., van der Ven-Jongekrijg J., Nieuwenhuijzen G.A.P., Sauerwein R.W., van der Meer J.W.M., Goris R.J.A. (1993). Cytokine Patterns in Patients after Major Vascular Surgery, Hemorrhagic Shock, and Severe Blunt Trauma Relation with Subsequent Adult Respiratory Distress Syndrome and Multiple Organ Failure. Ann. Surg..

[28] Halbgebauer R., Braun C.K., Denk S., Mayer B., Cinelli P., Radermacher P., Wanner G.A., Simmen H.-P., Gebhard F., Rittirsch D. (2018). Hemorrhagic shock drives glycocalyx, barrier and organ dysfunction early after polytrauma. J. Crit. Care.

[29] Davis J.W., Sue L.P., Dirks R.C., Kaups K.L., Kwok A.M., Wolfe M.M., Lilienstein J.T., Bilello J.F. (2020). Admission base deficit is superior to lactate in identifying shock and resuscitative needs in trauma patients. Am. J. Surg..

[30] Mestries J.C., Kruithof E.K., Gascon M.P., Herodin F., Agay D., Ythier A. (1994). In vivo modulation of coagulation and fibrinolysis by recombinant glycosylated human interleukin-6 in baboons. Eur. Cytokine Netw..

[31] Stouthard J.M., Levi M., E Hack C., Veenhof C.H., Romijn H.A., Sauerwein H.P., van der Poll T. (1996). Interleukin-6 stimulates coagulation, not fibrinolysis, in humans. Thromb. Haemost..

[32] Davenport R., Manson J., Deʼath H., Platton S., Coates A., Allard S., Hart D., Pearse R., Pasi K.J., Maccallum P. (2011). Functional definition and characterization of acute traumatic coagulopathy. Crit. Care Med..

[33] Schochl H., Cotton B., Inaba K., Nienaber U., Fischer H., Voelckel W., Solomon C. (2011). FIBTEM provides early prediction of massive transfusion in trauma. Crit. Care.

[34] Holcomb J.B., Tilley B.C., Baraniuk S., Fox E.E., Wade C.E., Podbielski J.M., del Junco D.J., Brasel K.J., Bulger E.M., Rachael A. (2015). Callcut Transfusion of plasma, platelets, and red blood cells in a 1:1:1 vs a 1:1:2 ratio and mortality in patients with severe trauma: The PROPPR randomized clinical trial. JAMA.

[35] Brøchner A.C., Toft P. (2009). Pathophysiology of the systemic inflammatory response after major accidental trauma. Scand. J. Trauma Resusc. Emerg. Med..

[36] Schöchl H., Nienaber U., Maegele M., Hochleitner G., Primavesi F., Steitz B., Arndt C., Hanke A., Voelckel W., Solomon C. (2011). Transfusion in trauma: Thromboelastometry-guided coagulation factor concentrate-based therapy versus standard fresh frozen plasma-based therapy. Crit. Care.

[37] Kaserer A., Casutt M., Sprengel K., Seifert B., Spahn D.R., Stein P. (2018). Comparison of two different coagulation algorithms on the use of allogenic blood products and coagulation factors in severely injured trauma patients: A retrospective, multicentre, observational study. Scand. J. Trauma Resusc. Emerg. Med..

[38] Innerhofer P., Fries D., Mittermayr M., Innerhofer N., von Langen D., Hell T., Gruber G., Schmid S., Friesenecker B., Lorenz I.H. (2017). Reversal of trauma-induced coagulopathy using first-line coagulation factor concentrates or fresh frozen plasma (RETIC): A single-centre, parallel-group, open-label, randomised trial. Lancet Haematol..

